# 2-[(1*Z*)-(9-Ethyl-9*H*-carbazol-3-yl)methyl­eneamino]-4,5,6,7-tetra­hydro-1-benzothio­phene-3-carbonitrile–benzene (2/1)

**DOI:** 10.1107/S1600536810014947

**Published:** 2010-04-28

**Authors:** Hoong-Kun Fun, Jia Hao Goh, Abdullah M. Asiri, Salman A. Khan, Khalid A. Khan

**Affiliations:** a X-ray Crystallography Unit, School of Physics, Universiti Sains Malaysia, 11800 USM, Penang, Malaysia; b Department of Chemistry, Faculty of Science, King Abdulaziz University, Jeddah, Saudi Arabia

## Abstract

In the title compound, 2C_24_H_21_N_3_S·C_6_H_6_, the two independent Schiff base mol­ecules (*A* and *B*) in the asymmetric unit differ in the orientation of the tetra­hydro­benzothio­phene ring system with respect to the carbazole ring system by 180° rotation about the C—C bond in the C—C=N—C linkage. The two mol­ecules also differ in the orientation of the ethyl groups [C—N—C—C torsion angle of 90.7 (3)° in mol­ecule *A*, and −79.4 (3)° in mol­ecule *B*]. In mol­ecule *B*, two methyl­ene C atoms of the cyclo­hexene ring are disordered over two sites with occupancies of 0.58 (1) and 0.42 (1). The cyclo­hexene rings in both mol­ecules adopt half-chair conformations. The dihedral angle between the thio­phene ring and the carbazole ring system is 8.07 (9)° in mol­ecule *A* [3.10 (9)° in mol­ecule *B*]. In the crystal structure, the independent mol­ecules are linked into dimers by inter­molecular C—H⋯N hydrogen bonds. In addition, C—H⋯π inter­actions are observed.

## Related literature

For biological and other applications of Schiff base compounds, see: Abu-Hussen (2006 ▶); Elerman *et al.* (2002 ▶); Panneerselvam *et al.* (2005 ▶); Walsh *et al.* (1996 ▶). For ring puckering parameters, see: Cremer & Pople (1975 ▶). For a related structure, see: Elerman & Elmali (1998 ▶).
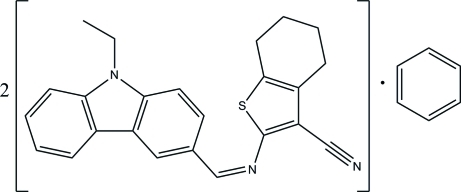


## Experimental

### 

#### Crystal data

2C_24_H_21_N_3_S·C_6_H_6_
*M* *_r_* = 845.10
Triclinic, 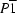

*a* = 11.4816 (1) Å
*b* = 13.7322 (2) Å
*c* = 14.8358 (2) Å
α = 81.841 (1)°
β = 77.083 (1)°
γ = 83.864 (1)°
*V* = 2250.00 (5) Å^3^
*Z* = 2
Mo *K*α radiation
μ = 0.16 mm^−1^
*T* = 293 K
0.45 × 0.15 × 0.07 mm

#### Data collection

Bruker SMART APEXII CCD area-detector diffractometer
Absorption correction: multi-scan (*SADABS*; Bruker, 2009 ▶) *T*_min_ = 0.931, *T*_max_ = 0.989
48693 measured reflections
13169 independent reflections
6227 reflections with *I* > 2σ(*I*)
*R*_int_ = 0.048

#### Refinement

*R*[*F* ^2^ > 2σ(*F* ^2^)] = 0.070
*wR*(*F* ^2^) = 0.167
*S* = 1.01
13169 reflections
580 parameters
63 restraints
H-atom parameters constrained
Δρ_max_ = 0.31 e Å^−3^
Δρ_min_ = −0.20 e Å^−3^

### 

Data collection: *APEX2* (Bruker, 2009 ▶); cell refinement: *SAINT* (Bruker, 2009 ▶); data reduction: *SAINT*; program(s) used to solve structure: *SHELXTL* (Sheldrick, 2008 ▶); program(s) used to refine structure: *SHELXTL*; molecular graphics: *SHELXTL*; software used to prepare material for publication: *SHELXTL* and *PLATON* (Spek, 2009 ▶).

## Supplementary Material

Crystal structure: contains datablocks global, I. DOI: 10.1107/S1600536810014947/ci5065sup1.cif

Structure factors: contains datablocks I. DOI: 10.1107/S1600536810014947/ci5065Isup2.hkl

Additional supplementary materials:  crystallographic information; 3D view; checkCIF report

## Figures and Tables

**Table 1:** Hydrogen-bond geometry (Å, °) *Cg*1, *Cg*2 and *Cg*3 are the centroids of the C1*B*–C6*B*, C7*A*–C12*A* and C14*A*–C16*A*/C21*A*/S1*A* rings, respectively.

*D*—H⋯*A*	*D*—H	H⋯*A*	*D*⋯*A*	*D*—H⋯*A*
C22*A*—H22*A*⋯N3*B*^i^	0.97	2.59	3.487 (3)	155
C11*A*—H11*A*⋯*Cg*1^ii^	0.93	2.65	3.499 (3)	153
C11*B*—H11*B*⋯*Cg*2^i^	0.93	2.82	3.725 (3)	166
C27—H27*A*⋯*Cg*3^iii^	0.93	2.71	3.625 (6)	169

Symmetry codes: (i) 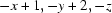; (ii) 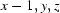; (iii) 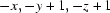.

**Fig. 1.**
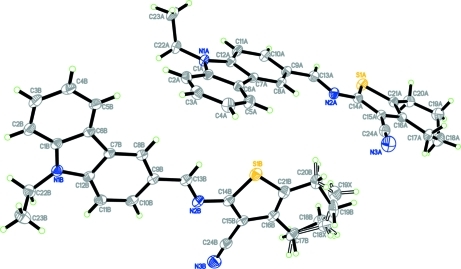
The asymmetric unit of the title compound. Displacement ellipsoids are drawn at the 20 % probability level. For clarity, the benzene solvent molecule is not shown.

**Fig. 2.**
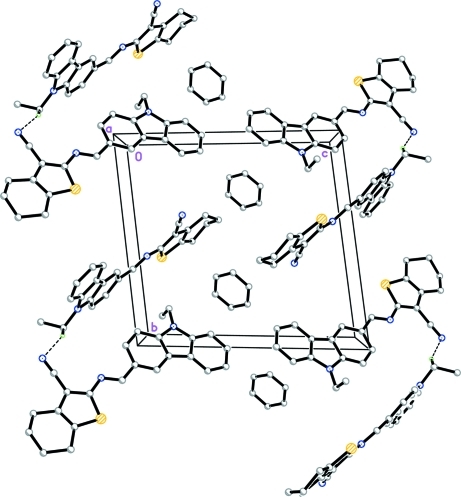
The crystal structure of the title compound, viewed along the a axis, showing the molecules being linked into dimers. Only the major disordered components are shown. H atoms not involved in intermolecular interactions (dashed lines) have been omitted for clarity.

## supplementary crystallographic information

### Comment

Schiff bases are an important class of compounds in medicinal and pharmaceutical field. They show biological applications including anti-bacterial (Abu-Hussen, 2006), anti-fungal (Panneerselvam *et al.*, 2005) and anti-tumor (Walsh *et al.*, 1996) activities. In the field of coordination chemistry, they are used extensively as ligands due to intramolecular hydrogen bonds between O and N atoms, which is applicable for the formation of metal complexes. Schiff bases are also used in the field of photochromism and thermochromism in the solid state (Elerman *et al.*, 2002). In this paper, we report the crystal structure of the title Schiff base compound.

The asymmetric unit of the title compound (Fig. 1) consists of two crystallographically independent Schiff base molecules and a benzene solvent molecule. The two independent main molecules differ in the orientation of the tetrahydrobenzothiophene ring system with respect to the 9*H*-carbazole ring system by 180° rotation about the C9—C13 bond. The conformation of the ethyl group (C22/C23) also differs in both molecules, with C1A—N1A—C22A—C23A torsion angle of 90.7 (3)° in molecule *A*, indicating (+)-anti-clinal conformation, whereas C1B—N1B—C22B—C23B torsion angle of -79.4 (3)° in molecule *B*, indicating (-)-syn-clinal conformation.

In molecule *A*, the cyclohexene ring (C16A-C21A) adopts a half-chair conformation, with puckering parameters of Q = 0.433 (3) Å, θ = 49.1 (4)° and φ = 145.4 (5)° (Cremer & Pople, 1975). In both the major and minor conformers of molecule *B*, the cyclohexene rings adopt half-chair conformations; the puckering parameters: Q = 0.508 (7) Å, θ = 50.0 (6)° and φ = 155.0 (7)° for the major conformer and Q = 0.467 (9) Å, θ = 130.4 (7)°, and φ = 330.0 (10)° for the minor conformer. The dihedral angle between the thiophene ring and the carbazole ring system is 8.07 (9)° in molecule *A* and 3.10 (9)° in molecule *B*. The bond lengths and angles are comparable to a related 4,5,6,7-tetrahydro-1-benzothiophene-3-carbonitrile structure (Elerman & Elmali, 1998).

In the crystal structure (Fig. 2), the benzene solvent molecule is not involved in intermolecular hydrogen bonding. The Schiff base molecules are linked into dimers by pairs of intermolecular C22A—H22A···N3B hydrogen bonds (Table 1). The crystal structure is further stabilized by weak intermolecular C—H···π interactions (Table 1).

### Experimental

A mixture of carbazolealdehyde (0.50 g, 0.0022 mol) and 2-amino-3-cyanobenzothiophene (0.38 g, 0.0022 mol) in methanol (15 ml) was refluxed for 5 h with stirring to give a light yellow precipitate. It was then filtered and washed with methanol to give the pure Schiff base. Good quality single crystals were recrystallized from a mixture of benzene, chloroform and methanol (4:4:2). (Yield 78 %, *M.p.* 468 K).

### Refinement

Atoms C18B and C19B are both disordered over two positions with occupancies of 0.58 (1) and 0.42 (1). In both disorder components, the C—C distances involving the disordered atoms were restrained to be equal. In the benzene solvent molecule, the C—C distances were restrained to 1.384 (3) Å. The U^ij^ components of atom C18X and all C atoms of the benzene solvent molecule were restrained to an approximate isotropic behaviour. H atoms were placed in their calculated positions [C–H = 0.93–97 Å] and refined using a riding model with *U*_iso_ = 1.2 or 1.5 *U*_eq_(C).

### Crystal data

**Table:**

2C_24_H_21_N_3_S·C_6_H_6_	*Z* = 2
*M**_r_* = 845.10	*F*(000) = 892
Triclinic, *P*1	*D*_x_ = 1.247 Mg m^−^^3^
Hall symbol: -P 1	Mo *K*α radiation, λ = 0.71073 Å
*a* = 11.4816 (1) Å	Cell parameters from 6974 reflections
*b* = 13.7322 (2) Å	θ = 2.4–21.2°
*c* = 14.8358 (2) Å	µ = 0.16 mm^−^^1^
α = 81.841 (1)°	*T* = 293 K
β = 77.083 (1)°	Plate, yellow
γ = 83.864 (1)°	0.45 × 0.15 × 0.07 mm
*V* = 2250.00 (5) Å^3^	

### Data collection

**Table:**

Bruker SMART APEXII CCD area-detector diffractometer	13169 independent reflections
Radiation source: fine-focus sealed tube	6227 reflections with *I* > 2σ(*I*)
graphite	*R*_int_ = 0.048
φ and ω scans	θ_max_ = 30.1°, θ_min_ = 1.8°
Absorption correction: multi-scan (*SADABS*; Bruker, 2009)	*h* = −16→16
*T*_min_ = 0.931, *T*_max_ = 0.989	*k* = −19→19
48693 measured reflections	*l* = −20→20

### Refinement

**Table:**

Refinement on *F*^2^	Primary atom site location: structure-invariant direct methods
Least-squares matrix: full	Secondary atom site location: difference Fourier map
*R*[*F*^2^ > 2σ(*F*^2^)] = 0.070	Hydrogen site location: inferred from neighbouring sites
*wR*(*F*^2^) = 0.167	H-atom parameters constrained
*S* = 1.01	*w* = 1/[σ^2^(*F*_o_^2^) + (0.0574*P*)^2^ + 0.4624*P*] where *P* = (*F*_o_^2^ + 2*F*_c_^2^)/3
13169 reflections	(Δ/σ)_max_ = 0.001
580 parameters	Δρ_max_ = 0.31 e Å^−^^3^
63 restraints	Δρ_min_ = −0.20 e Å^−^^3^

### Special details

**Table:**

Geometry. All esds (except the esd in the dihedral angle between two l.s. planes) are estimated using the full covariance matrix. The cell esds are taken into account individually in the estimation of esds in distances, angles and torsion angles; correlations between esds in cell parameters are only used when they are defined by crystal symmetry. An approximate (isotropic) treatment of cell esds is used for estimating esds involving l.s. planes.
Refinement. Refinement of F^2^ against ALL reflections. The weighted R-factor wR and goodness of fit S are based on F^2^, conventional R-factors R are based on F, with F set to zero for negative F^2^. The threshold expression of F^2^ > 2sigma(F^2^) is used only for calculating R-factors(gt) etc. and is not relevant to the choice of reflections for refinement. R-factors based on F^2^ are statistically about twice as large as those based on F, and R- factors based on ALL data will be even larger.

### Fractional atomic coordinates and isotropic or equivalent isotropic displacement parameters (Å^2^)

**Table:**

	*x*	*y*	*z*	*U*_iso_*/*U*_eq_	Occ. (<1)
S1A	−0.28193 (6)	0.58795 (5)	0.14257 (5)	0.0645 (2)	
N1A	0.35074 (17)	0.81675 (13)	−0.21845 (13)	0.0532 (5)	
N2A	−0.03256 (16)	0.58008 (14)	0.08737 (14)	0.0554 (5)	
N3A	0.0583 (2)	0.3886 (2)	0.2688 (2)	0.0964 (8)	
C1A	0.4457 (2)	0.75882 (16)	−0.18956 (15)	0.0496 (5)	
C2A	0.5683 (2)	0.76344 (19)	−0.22354 (18)	0.0622 (7)	
H2A	0.5983	0.8120	−0.2709	0.075*	
C3A	0.6437 (2)	0.6938 (2)	−0.1847 (2)	0.0693 (7)	
H3A	0.7259	0.6942	−0.2075	0.083*	
C4A	0.6002 (2)	0.6226 (2)	−0.1121 (2)	0.0718 (7)	
H4A	0.6537	0.5773	−0.0864	0.086*	
C5A	0.4785 (2)	0.61827 (18)	−0.07770 (18)	0.0630 (7)	
H5A	0.4497	0.5709	−0.0287	0.076*	
C6A	0.39982 (19)	0.68584 (16)	−0.11748 (15)	0.0481 (5)	
C7A	0.27086 (19)	0.69989 (15)	−0.10253 (14)	0.0452 (5)	
C8A	0.17748 (19)	0.65416 (16)	−0.04151 (15)	0.0492 (5)	
H8A	0.1932	0.6022	0.0027	0.059*	
C9A	0.0596 (2)	0.68660 (16)	−0.04674 (15)	0.0504 (5)	
C10A	0.0376 (2)	0.76495 (18)	−0.11427 (17)	0.0603 (6)	
H10A	−0.0413	0.7849	−0.1182	0.072*	
C11A	0.1282 (2)	0.81317 (17)	−0.17470 (16)	0.0573 (6)	
H11A	0.1119	0.8654	−0.2185	0.069*	
C12A	0.2451 (2)	0.78087 (16)	−0.16790 (15)	0.0478 (5)	
C13A	−0.0413 (2)	0.64287 (17)	0.01630 (17)	0.0559 (6)	
H13A	−0.1173	0.6615	0.0045	0.067*	
C14A	−0.13382 (19)	0.54642 (16)	0.14786 (16)	0.0514 (6)	
C15A	−0.13252 (19)	0.47652 (16)	0.22303 (16)	0.0488 (5)	
C16A	−0.2488 (2)	0.45626 (16)	0.27752 (15)	0.0486 (5)	
C17A	−0.2730 (2)	0.3862 (2)	0.36522 (18)	0.0672 (7)	
H17A	−0.2154	0.3924	0.4025	0.081*	
H17B	−0.2633	0.3191	0.3498	0.081*	
C18A	−0.3980 (3)	0.4065 (2)	0.4210 (2)	0.0870 (9)	
H18A	−0.4179	0.3506	0.4679	0.104*	
H18B	−0.3997	0.4637	0.4529	0.104*	
C19A	−0.4892 (3)	0.4243 (3)	0.3640 (2)	0.0874 (9)	
H19A	−0.5669	0.4367	0.4044	0.105*	
H19B	−0.4917	0.3650	0.3361	0.105*	
C20A	−0.4682 (2)	0.5109 (2)	0.28643 (19)	0.0695 (7)	
H20A	−0.5132	0.5049	0.2396	0.083*	
H20B	−0.4961	0.5725	0.3120	0.083*	
C21A	−0.3371 (2)	0.51130 (17)	0.24260 (16)	0.0534 (6)	
C22A	0.3612 (2)	0.90256 (18)	−0.28954 (17)	0.0651 (7)	
H22A	0.4326	0.9343	−0.2893	0.078*	
H22B	0.2926	0.9494	−0.2737	0.078*	
C23A	0.3678 (3)	0.8773 (2)	−0.38548 (18)	0.0853 (9)	
H23A	0.3759	0.9362	−0.4291	0.128*	
H23B	0.2959	0.8483	−0.3870	0.128*	
H23C	0.4357	0.8313	−0.4019	0.128*	
C24A	−0.0248 (2)	0.42838 (19)	0.24646 (18)	0.0624 (7)	
S1B	0.38291 (6)	0.72056 (5)	0.14806 (5)	0.0707 (2)	
N1B	0.91999 (18)	1.09596 (14)	−0.17385 (13)	0.0557 (5)	
N2B	0.50847 (18)	0.88652 (15)	0.13816 (13)	0.0599 (5)	
N3B	0.3760 (2)	0.9764 (2)	0.36567 (18)	0.0917 (8)	
C1B	0.9522 (2)	1.04208 (17)	−0.24963 (16)	0.0530 (6)	
C2B	1.0415 (2)	1.05656 (19)	−0.32907 (17)	0.0618 (7)	
H2B	1.0898	1.1089	−0.3387	0.074*	
C3B	1.0561 (2)	0.9905 (2)	−0.39332 (18)	0.0706 (7)	
H3B	1.1156	0.9984	−0.4472	0.085*	
C4B	0.9841 (2)	0.9125 (2)	−0.37945 (17)	0.0703 (7)	
H4B	0.9961	0.8692	−0.4241	0.084*	
C5B	0.8949 (2)	0.8980 (2)	−0.30040 (16)	0.0605 (6)	
H5B	0.8467	0.8457	−0.2917	0.073*	
C6B	0.87815 (19)	0.96298 (17)	−0.23398 (15)	0.0493 (5)	
C7B	0.79972 (19)	0.96862 (16)	−0.14364 (15)	0.0482 (5)	
C8B	0.7109 (2)	0.91230 (17)	−0.08940 (15)	0.0529 (6)	
H8B	0.6898	0.8582	−0.1118	0.063*	
C9B	0.6528 (2)	0.93600 (17)	−0.00138 (16)	0.0535 (6)	
C10B	0.6838 (2)	1.01967 (18)	0.03051 (16)	0.0593 (6)	
H10B	0.6439	1.0362	0.0888	0.071*	
C11B	0.7708 (2)	1.07744 (18)	−0.02177 (17)	0.0594 (6)	
H11B	0.7902	1.1324	0.0003	0.071*	
C12B	0.8295 (2)	1.05139 (17)	−0.10915 (16)	0.0517 (6)	
C13B	0.5630 (2)	0.87384 (19)	0.05443 (17)	0.0596 (6)	
H13B	0.5435	0.8217	0.0284	0.071*	
C14B	0.4238 (2)	0.82359 (18)	0.18658 (16)	0.0574 (6)	
C15B	0.3571 (2)	0.83434 (19)	0.27491 (16)	0.0574 (6)	
C16B	0.2759 (2)	0.7603 (2)	0.31164 (17)	0.0590 (6)	
C17B	0.1949 (2)	0.7533 (2)	0.40691 (18)	0.0787 (8)	
H17C	0.2435	0.7324	0.4529	0.094*	0.580 (10)
H17D	0.1577	0.8186	0.4178	0.094*	0.580 (10)
H17E	0.1262	0.8000	0.4088	0.094*	0.420 (10)
H17F	0.2372	0.7640	0.4532	0.094*	0.420 (10)
C18B	0.1000 (5)	0.6851 (5)	0.4213 (6)	0.094 (3)	0.580 (10)
H18C	0.0360	0.7143	0.3907	0.113*	0.580 (10)
H18D	0.0667	0.6690	0.4872	0.113*	0.580 (10)
C19B	0.1608 (7)	0.5936 (5)	0.3786 (4)	0.097 (3)	0.580 (10)
H19C	0.1046	0.5428	0.3946	0.117*	0.580 (10)
H19D	0.2281	0.5699	0.4074	0.117*	0.580 (10)
C18X	0.1499 (9)	0.6467 (6)	0.4240 (7)	0.082 (3)	0.420 (10)
H18E	0.2161	0.6012	0.4376	0.098*	0.420 (10)
H18F	0.0876	0.6437	0.4803	0.098*	0.420 (10)
C19X	0.1007 (7)	0.6051 (8)	0.3504 (5)	0.085 (3)	0.420 (10)
H19E	0.0767	0.5387	0.3719	0.102*	0.420 (10)
H19F	0.0332	0.6468	0.3338	0.102*	0.420 (10)
C20B	0.2076 (3)	0.6061 (2)	0.2700 (2)	0.0834 (9)	
H20C	0.2568	0.5479	0.2500	0.100*	0.580 (10)
H20D	0.1414	0.6179	0.2381	0.100*	0.580 (10)
H20E	0.2600	0.5483	0.2810	0.100*	0.420 (10)
H20F	0.1822	0.6014	0.2135	0.100*	0.420 (10)
C21B	0.2802 (2)	0.6939 (2)	0.25154 (19)	0.0662 (7)	
C22B	0.9866 (2)	1.17511 (18)	−0.1602 (2)	0.0675 (7)	
H22C	0.9346	1.2166	−0.1176	0.081*	
H22D	1.0111	1.2156	−0.2193	0.081*	
C23B	1.0961 (3)	1.1365 (2)	−0.1218 (2)	0.0892 (9)	
H23D	1.1388	1.1909	−0.1160	0.134*	
H23E	1.1473	1.0946	−0.1633	0.134*	
H23F	1.0720	1.0995	−0.0617	0.134*	
C24B	0.3697 (2)	0.9139 (2)	0.32369 (18)	0.0665 (7)	
C25	0.3449 (4)	0.1706 (3)	0.5697 (4)	0.1256 (15)	
H25A	0.3955	0.1138	0.5770	0.151*	
C26	0.3230 (4)	0.2345 (4)	0.6346 (3)	0.1390 (16)	
H26A	0.3558	0.2215	0.6874	0.167*	
C27	0.2512 (5)	0.3183 (4)	0.6194 (4)	0.1336 (17)	
H27A	0.2339	0.3622	0.6638	0.160*	
C28	0.2037 (4)	0.3403 (3)	0.5419 (4)	0.1294 (17)	
H28A	0.1579	0.3994	0.5324	0.155*	
C29	0.2247 (4)	0.2739 (4)	0.4784 (3)	0.1280 (15)	
H29A	0.1923	0.2866	0.4255	0.154*	
C30	0.2938 (4)	0.1888 (4)	0.4942 (3)	0.1156 (14)	
H30A	0.3062	0.1423	0.4525	0.139*	

### Atomic displacement parameters (Å^2^)

**Table:**

	*U*^11^	*U*^22^	*U*^33^	*U*^12^	*U*^13^	*U*^23^
S1A	0.0501 (4)	0.0724 (4)	0.0636 (4)	0.0010 (3)	−0.0070 (3)	0.0043 (3)
N1A	0.0549 (12)	0.0531 (11)	0.0460 (11)	−0.0064 (9)	−0.0053 (9)	0.0061 (9)
N2A	0.0508 (11)	0.0565 (12)	0.0528 (12)	−0.0063 (9)	0.0015 (9)	−0.0040 (10)
N3A	0.0632 (16)	0.099 (2)	0.122 (2)	0.0068 (14)	−0.0265 (16)	0.0024 (17)
C1A	0.0507 (13)	0.0514 (13)	0.0442 (13)	−0.0043 (10)	−0.0036 (11)	−0.0068 (10)
C2A	0.0581 (15)	0.0664 (16)	0.0602 (16)	−0.0146 (13)	−0.0042 (13)	−0.0071 (13)
C3A	0.0501 (14)	0.0766 (18)	0.0796 (19)	−0.0084 (13)	−0.0039 (14)	−0.0170 (16)
C4A	0.0542 (16)	0.0708 (18)	0.087 (2)	0.0061 (13)	−0.0172 (15)	−0.0039 (16)
C5A	0.0568 (15)	0.0598 (15)	0.0654 (17)	0.0018 (12)	−0.0084 (13)	0.0039 (13)
C6A	0.0492 (13)	0.0477 (12)	0.0443 (13)	0.0010 (10)	−0.0049 (10)	−0.0069 (10)
C7A	0.0499 (12)	0.0441 (12)	0.0388 (12)	0.0029 (9)	−0.0065 (10)	−0.0051 (10)
C8A	0.0545 (14)	0.0456 (12)	0.0413 (12)	0.0009 (10)	−0.0029 (11)	0.0001 (10)
C9A	0.0498 (13)	0.0515 (13)	0.0450 (13)	0.0009 (10)	−0.0031 (11)	−0.0046 (11)
C10A	0.0498 (14)	0.0666 (16)	0.0590 (15)	0.0027 (12)	−0.0087 (12)	0.0003 (13)
C11A	0.0585 (15)	0.0568 (14)	0.0507 (14)	0.0035 (12)	−0.0111 (12)	0.0068 (11)
C12A	0.0520 (13)	0.0479 (12)	0.0393 (12)	−0.0008 (10)	−0.0035 (10)	−0.0026 (10)
C13A	0.0476 (13)	0.0606 (15)	0.0560 (15)	−0.0020 (11)	−0.0038 (12)	−0.0082 (12)
C14A	0.0460 (12)	0.0533 (13)	0.0516 (14)	−0.0049 (10)	−0.0014 (11)	−0.0090 (11)
C15A	0.0439 (12)	0.0499 (13)	0.0516 (14)	−0.0021 (10)	−0.0063 (11)	−0.0094 (11)
C16A	0.0480 (13)	0.0509 (13)	0.0454 (13)	−0.0054 (10)	−0.0038 (11)	−0.0092 (10)
C17A	0.0643 (16)	0.0699 (17)	0.0602 (16)	−0.0107 (13)	−0.0016 (13)	0.0018 (13)
C18A	0.0717 (19)	0.101 (2)	0.073 (2)	−0.0109 (16)	0.0082 (17)	0.0050 (17)
C19A	0.0588 (17)	0.122 (3)	0.075 (2)	−0.0309 (17)	0.0083 (16)	−0.0096 (19)
C20A	0.0441 (13)	0.0883 (19)	0.0717 (18)	−0.0063 (13)	0.0000 (13)	−0.0132 (15)
C21A	0.0463 (13)	0.0589 (14)	0.0524 (14)	−0.0078 (11)	−0.0023 (11)	−0.0086 (11)
C22A	0.0695 (17)	0.0569 (15)	0.0603 (16)	−0.0095 (12)	−0.0058 (13)	0.0121 (12)
C23A	0.111 (2)	0.0760 (19)	0.0556 (17)	−0.0056 (17)	−0.0047 (16)	0.0147 (14)
C24A	0.0514 (15)	0.0620 (16)	0.0688 (17)	−0.0038 (12)	−0.0055 (13)	−0.0031 (13)
S1B	0.0759 (5)	0.0701 (4)	0.0595 (4)	−0.0001 (3)	−0.0028 (4)	−0.0085 (3)
N1B	0.0635 (12)	0.0509 (11)	0.0520 (12)	−0.0042 (9)	−0.0134 (10)	−0.0024 (9)
N2B	0.0579 (12)	0.0684 (13)	0.0458 (12)	0.0043 (10)	−0.0037 (10)	−0.0002 (10)
N3B	0.0908 (19)	0.112 (2)	0.0738 (17)	−0.0163 (16)	−0.0070 (14)	−0.0249 (16)
C1B	0.0545 (14)	0.0568 (14)	0.0454 (13)	0.0036 (11)	−0.0163 (11)	0.0045 (11)
C2B	0.0590 (15)	0.0697 (16)	0.0511 (15)	−0.0039 (12)	−0.0111 (12)	0.0101 (13)
C3B	0.0669 (17)	0.094 (2)	0.0426 (14)	−0.0024 (15)	−0.0040 (13)	0.0057 (14)
C4B	0.0740 (18)	0.092 (2)	0.0437 (14)	0.0003 (16)	−0.0083 (13)	−0.0153 (14)
C5B	0.0610 (15)	0.0772 (17)	0.0442 (14)	−0.0056 (13)	−0.0104 (12)	−0.0112 (12)
C6B	0.0484 (13)	0.0581 (14)	0.0404 (12)	0.0040 (11)	−0.0133 (10)	−0.0025 (11)
C7B	0.0492 (13)	0.0524 (13)	0.0423 (12)	0.0044 (10)	−0.0125 (11)	−0.0048 (10)
C8B	0.0549 (14)	0.0572 (14)	0.0464 (13)	−0.0001 (11)	−0.0112 (11)	−0.0079 (11)
C9B	0.0531 (14)	0.0599 (14)	0.0447 (13)	0.0050 (11)	−0.0098 (11)	−0.0045 (11)
C10B	0.0674 (16)	0.0650 (16)	0.0416 (13)	0.0082 (13)	−0.0078 (12)	−0.0099 (12)
C11B	0.0715 (17)	0.0567 (15)	0.0525 (15)	0.0037 (12)	−0.0169 (13)	−0.0154 (12)
C12B	0.0573 (14)	0.0513 (13)	0.0450 (13)	0.0049 (11)	−0.0164 (11)	0.0014 (11)
C13B	0.0575 (15)	0.0661 (16)	0.0517 (15)	0.0030 (12)	−0.0097 (12)	−0.0045 (12)
C14B	0.0547 (14)	0.0641 (15)	0.0488 (14)	0.0056 (12)	−0.0101 (12)	0.0002 (12)
C15B	0.0531 (14)	0.0705 (16)	0.0451 (14)	0.0077 (12)	−0.0115 (12)	−0.0023 (12)
C16B	0.0460 (13)	0.0734 (17)	0.0496 (14)	0.0070 (12)	−0.0062 (11)	0.0039 (13)
C17B	0.0623 (17)	0.102 (2)	0.0585 (17)	0.0043 (16)	−0.0010 (14)	0.0067 (16)
C18B	0.040 (3)	0.157 (7)	0.080 (4)	−0.020 (4)	−0.004 (3)	−0.003 (4)
C19B	0.061 (4)	0.065 (4)	0.149 (8)	−0.018 (4)	0.002 (5)	0.016 (4)
C18X	0.038 (5)	0.145 (8)	0.059 (5)	−0.020 (5)	−0.002 (4)	−0.004 (5)
C19X	0.051 (5)	0.101 (6)	0.097 (7)	−0.013 (5)	0.005 (5)	−0.016 (5)
C20B	0.078 (2)	0.076 (2)	0.089 (2)	−0.0112 (15)	−0.0121 (17)	0.0067 (17)
C21B	0.0565 (15)	0.0678 (17)	0.0664 (17)	0.0037 (13)	−0.0087 (13)	0.0050 (14)
C22B	0.0768 (18)	0.0512 (15)	0.0759 (18)	−0.0068 (13)	−0.0204 (15)	−0.0041 (13)
C23B	0.081 (2)	0.086 (2)	0.111 (3)	0.0008 (16)	−0.0391 (19)	−0.0243 (19)
C24B	0.0595 (16)	0.087 (2)	0.0484 (15)	−0.0008 (14)	−0.0069 (13)	−0.0038 (15)
C25	0.096 (3)	0.097 (3)	0.156 (4)	−0.003 (2)	0.014 (3)	0.009 (3)
C26	0.114 (3)	0.178 (5)	0.132 (4)	−0.042 (3)	−0.026 (3)	−0.022 (4)
C27	0.128 (4)	0.118 (4)	0.162 (4)	−0.040 (3)	0.005 (3)	−0.079 (3)
C28	0.080 (3)	0.086 (3)	0.196 (5)	−0.003 (2)	0.011 (3)	0.008 (3)
C29	0.093 (3)	0.183 (5)	0.098 (3)	−0.044 (3)	−0.005 (2)	0.017 (3)
C30	0.119 (3)	0.121 (3)	0.098 (3)	−0.038 (3)	0.032 (2)	−0.051 (3)

### Geometric parameters (Å, °)

**Table:**

S1A—C21A	1.729 (2)	C3B—C4B	1.388 (4)
S1A—C14A	1.750 (2)	C3B—H3B	0.93
N1A—C12A	1.376 (3)	C4B—C5B	1.380 (3)
N1A—C1A	1.390 (3)	C4B—H4B	0.93
N1A—C22A	1.462 (3)	C5B—C6B	1.390 (3)
N2A—C13A	1.279 (3)	C5B—H5B	0.93
N2A—C14A	1.379 (3)	C6B—C7B	1.445 (3)
N3A—C24A	1.139 (3)	C7B—C8B	1.382 (3)
C1A—C2A	1.390 (3)	C7B—C12B	1.413 (3)
C1A—C6A	1.408 (3)	C8B—C9B	1.393 (3)
C2A—C3A	1.373 (4)	C8B—H8B	0.93
C2A—H2A	0.93	C9B—C10B	1.409 (3)
C3A—C4A	1.388 (4)	C9B—C13B	1.443 (3)
C3A—H3A	0.93	C10B—C11B	1.370 (3)
C4A—C5A	1.381 (3)	C10B—H10B	0.93
C4A—H4A	0.93	C11B—C12B	1.398 (3)
C5A—C6A	1.389 (3)	C11B—H11B	0.93
C5A—H5A	0.93	C13B—H13B	0.93
C6A—C7A	1.443 (3)	C14B—C15B	1.381 (3)
C7A—C8A	1.383 (3)	C15B—C16B	1.421 (3)
C7A—C12A	1.420 (3)	C15B—C24B	1.430 (4)
C8A—C9A	1.394 (3)	C16B—C21B	1.353 (4)
C8A—H8A	0.93	C16B—C17B	1.505 (3)
C9A—C10A	1.405 (3)	C17B—C18B	1.472 (5)
C9A—C13A	1.444 (3)	C17B—C18X	1.573 (7)
C10A—C11A	1.374 (3)	C17B—H17C	0.97
C10A—H10A	0.93	C17B—H17D	0.97
C11A—C12A	1.388 (3)	C17B—H17E	0.96
C11A—H11A	0.93	C17B—H17F	0.96
C13A—H13A	0.93	C18B—C19B	1.520 (6)
C14A—C15A	1.366 (3)	C18B—H18C	0.97
C15A—C24A	1.428 (4)	C18B—H18D	0.97
C15A—C16A	1.430 (3)	C19B—C20B	1.571 (6)
C16A—C21A	1.352 (3)	C19B—H19C	0.97
C16A—C17A	1.495 (3)	C19B—H19D	0.97
C17A—C18A	1.506 (4)	C18X—C19X	1.535 (7)
C17A—H17A	0.97	C18X—H18E	0.97
C17A—H17B	0.97	C18X—H18F	0.97
C18A—C19A	1.467 (4)	C19X—C20B	1.508 (6)
C18A—H18A	0.97	C19X—H19E	0.97
C18A—H18B	0.97	C19X—H19F	0.97
C19A—C20A	1.531 (4)	C20B—C21B	1.497 (4)
C19A—H19A	0.97	C20B—H20C	0.97
C19A—H19B	0.97	C20B—H20D	0.97
C20A—C21A	1.501 (3)	C20B—H20E	0.96
C20A—H20A	0.97	C20B—H20F	0.96
C20A—H20B	0.97	C22B—C23B	1.510 (4)
C22A—C23A	1.496 (4)	C22B—H22C	0.97
C22A—H22A	0.97	C22B—H22D	0.97
C22A—H22B	0.97	C23B—H23D	0.96
C23A—H23A	0.96	C23B—H23E	0.96
C23A—H23B	0.96	C23B—H23F	0.96
C23A—H23C	0.96	C25—C26	1.358 (7)
S1B—C21B	1.734 (3)	C25—C30	1.358 (7)
S1B—C14B	1.743 (3)	C25—H25A	0.93
N1B—C12B	1.378 (3)	C26—C27	1.364 (8)
N1B—C1B	1.393 (3)	C26—H26A	0.93
N1B—C22B	1.455 (3)	C27—C28	1.363 (8)
N2B—C13B	1.287 (3)	C27—H27A	0.93
N2B—C14B	1.378 (3)	C28—C29	1.368 (7)
N3B—C24B	1.146 (3)	C28—H28A	0.93
C1B—C2B	1.385 (3)	C29—C30	1.363 (7)
C1B—C6B	1.412 (3)	C29—H29A	0.93
C2B—C3B	1.379 (4)	C30—H30A	0.93
C2B—H2B	0.93		
			
C21A—S1A—C14A	91.75 (11)	C7B—C8B—H8B	119.8
C12A—N1A—C1A	108.65 (18)	C9B—C8B—H8B	119.8
C12A—N1A—C22A	125.6 (2)	C8B—C9B—C10B	119.1 (2)
C1A—N1A—C22A	125.74 (19)	C8B—C9B—C13B	118.9 (2)
C13A—N2A—C14A	120.6 (2)	C10B—C9B—C13B	122.1 (2)
N1A—C1A—C2A	129.5 (2)	C11B—C10B—C9B	122.0 (2)
N1A—C1A—C6A	109.02 (19)	C11B—C10B—H10B	119.0
C2A—C1A—C6A	121.5 (2)	C9B—C10B—H10B	119.0
C3A—C2A—C1A	117.6 (2)	C10B—C11B—C12B	118.1 (2)
C3A—C2A—H2A	121.2	C10B—C11B—H11B	121.0
C1A—C2A—H2A	121.2	C12B—C11B—H11B	121.0
C2A—C3A—C4A	121.7 (2)	N1B—C12B—C11B	129.4 (2)
C2A—C3A—H3A	119.1	N1B—C12B—C7B	109.3 (2)
C4A—C3A—H3A	119.1	C11B—C12B—C7B	121.3 (2)
C5A—C4A—C3A	120.8 (3)	N2B—C13B—C9B	123.7 (2)
C5A—C4A—H4A	119.6	N2B—C13B—H13B	118.1
C3A—C4A—H4A	119.6	C9B—C13B—H13B	118.1
C4A—C5A—C6A	118.9 (2)	N2B—C14B—C15B	123.8 (2)
C4A—C5A—H5A	120.6	N2B—C14B—S1B	126.62 (18)
C6A—C5A—H5A	120.6	C15B—C14B—S1B	109.55 (19)
C5A—C6A—C1A	119.5 (2)	C14B—C15B—C16B	114.1 (2)
C5A—C6A—C7A	133.6 (2)	C14B—C15B—C24B	122.1 (2)
C1A—C6A—C7A	106.86 (19)	C16B—C15B—C24B	123.8 (2)
C8A—C7A—C12A	119.4 (2)	C21B—C16B—C15B	112.4 (2)
C8A—C7A—C6A	134.4 (2)	C21B—C16B—C17B	122.7 (2)
C12A—C7A—C6A	106.16 (18)	C15B—C16B—C17B	125.0 (3)
C7A—C8A—C9A	119.5 (2)	C18B—C17B—C16B	114.8 (4)
C7A—C8A—H8A	120.2	C16B—C17B—C18X	105.1 (4)
C9A—C8A—H8A	120.2	C18B—C17B—H17C	108.6
C8A—C9A—C10A	119.5 (2)	C16B—C17B—H17C	108.6
C8A—C9A—C13A	121.9 (2)	C18X—C17B—H17C	88.7
C10A—C9A—C13A	118.6 (2)	C18B—C17B—H17D	108.6
C11A—C10A—C9A	122.5 (2)	C16B—C17B—H17D	108.6
C11A—C10A—H10A	118.8	C18X—C17B—H17D	134.9
C9A—C10A—H10A	118.8	H17C—C17B—H17D	107.5
C10A—C11A—C12A	117.4 (2)	C18B—C17B—H17E	80.2
C10A—C11A—H11A	121.3	C16B—C17B—H17E	111.5
C12A—C11A—H11A	121.3	C18X—C17B—H17E	108.0
N1A—C12A—C11A	129.0 (2)	H17C—C17B—H17E	130.0
N1A—C12A—C7A	109.28 (19)	C18B—C17B—H17F	125.3
C11A—C12A—C7A	121.7 (2)	C16B—C17B—H17F	110.9
N2A—C13A—C9A	123.9 (2)	C18X—C17B—H17F	112.0
N2A—C13A—H13A	118.0	H17D—C17B—H17F	82.7
C9A—C13A—H13A	118.0	H17E—C17B—H17F	109.3
C15A—C14A—N2A	124.4 (2)	C17B—C18B—C19B	105.3 (5)
C15A—C14A—S1A	109.76 (16)	C17B—C18B—H18C	110.7
N2A—C14A—S1A	125.85 (18)	C19B—C18B—H18C	110.7
C14A—C15A—C24A	123.3 (2)	C17B—C18B—H18D	110.7
C14A—C15A—C16A	114.2 (2)	C19B—C18B—H18D	110.7
C24A—C15A—C16A	122.5 (2)	H18C—C18B—H18D	108.8
C21A—C16A—C15A	112.0 (2)	C18B—C19B—C20B	116.5 (6)
C21A—C16A—C17A	122.6 (2)	C18B—C19B—H19C	108.2
C15A—C16A—C17A	125.3 (2)	C20B—C19B—H19C	108.2
C16A—C17A—C18A	111.3 (2)	C18B—C19B—H19D	108.2
C16A—C17A—H17A	109.4	C20B—C19B—H19D	108.2
C18A—C17A—H17A	109.4	H19C—C19B—H19D	107.3
C16A—C17A—H17B	109.4	C19X—C18X—C17B	121.8 (7)
C18A—C17A—H17B	109.4	C19X—C18X—H18E	106.9
H17A—C17A—H17B	108.0	C17B—C18X—H18E	106.9
C19A—C18A—C17A	113.3 (3)	C19X—C18X—H18F	106.9
C19A—C18A—H18A	108.9	C17B—C18X—H18F	106.9
C17A—C18A—H18A	108.9	H18E—C18X—H18F	106.7
C19A—C18A—H18B	108.9	C20B—C19X—C18X	101.7 (7)
C17A—C18A—H18B	108.9	C20B—C19X—H19E	111.4
H18A—C18A—H18B	107.7	C18X—C19X—H19E	111.4
C18A—C19A—C20A	114.3 (2)	C20B—C19X—H19F	111.4
C18A—C19A—H19A	108.7	C18X—C19X—H19F	111.4
C20A—C19A—H19A	108.7	H19E—C19X—H19F	109.3
C18A—C19A—H19B	108.7	C21B—C20B—C19X	116.9 (4)
C20A—C19A—H19B	108.7	C21B—C20B—C19B	103.9 (4)
H19A—C19A—H19B	107.6	C21B—C20B—H20C	111.0
C21A—C20A—C19A	109.7 (2)	C19X—C20B—H20C	124.8
C21A—C20A—H20A	109.7	C19B—C20B—H20C	111.0
C19A—C20A—H20A	109.7	C21B—C20B—H20D	111.0
C21A—C20A—H20B	109.7	C19X—C20B—H20D	78.1
C19A—C20A—H20B	109.7	C19B—C20B—H20D	111.0
H20A—C20A—H20B	108.2	H20C—C20B—H20D	109.0
C16A—C21A—C20A	124.9 (2)	C21B—C20B—H20E	107.9
C16A—C21A—S1A	112.23 (17)	C19X—C20B—H20E	108.1
C20A—C21A—S1A	122.78 (19)	C19B—C20B—H20E	85.4
N1A—C22A—C23A	113.3 (2)	H20D—C20B—H20E	131.9
N1A—C22A—H22A	108.9	C21B—C20B—H20F	106.7
C23A—C22A—H22A	108.9	C19X—C20B—H20F	109.8
N1A—C22A—H22B	108.9	C19B—C20B—H20F	141.1
C23A—C22A—H22B	108.9	H20C—C20B—H20F	79.6
H22A—C22A—H22B	107.7	H20E—C20B—H20F	107.0
C22A—C23A—H23A	109.5	C16B—C21B—C20B	125.5 (2)
C22A—C23A—H23B	109.5	C16B—C21B—S1B	112.0 (2)
H23A—C23A—H23B	109.5	C20B—C21B—S1B	122.5 (2)
C22A—C23A—H23C	109.5	N1B—C22B—C23B	112.2 (2)
H23A—C23A—H23C	109.5	N1B—C22B—H22C	109.2
H23B—C23A—H23C	109.5	C23B—C22B—H22C	109.2
N3A—C24A—C15A	177.1 (3)	N1B—C22B—H22D	109.2
C21B—S1B—C14B	91.95 (13)	C23B—C22B—H22D	109.2
C12B—N1B—C1B	108.69 (19)	H22C—C22B—H22D	107.9
C12B—N1B—C22B	126.4 (2)	C22B—C23B—H23D	109.5
C1B—N1B—C22B	124.1 (2)	C22B—C23B—H23E	109.5
C13B—N2B—C14B	120.0 (2)	H23D—C23B—H23E	109.5
C2B—C1B—N1B	129.3 (2)	C22B—C23B—H23F	109.5
C2B—C1B—C6B	121.9 (2)	H23D—C23B—H23F	109.5
N1B—C1B—C6B	108.8 (2)	H23E—C23B—H23F	109.5
C3B—C2B—C1B	117.5 (2)	N3B—C24B—C15B	177.2 (3)
C3B—C2B—H2B	121.3	C26—C25—C30	120.7 (4)
C1B—C2B—H2B	121.3	C26—C25—H25A	119.6
C2B—C3B—C4B	121.5 (2)	C30—C25—H25A	119.6
C2B—C3B—H3B	119.2	C25—C26—C27	117.5 (4)
C4B—C3B—H3B	119.2	C25—C26—H26A	121.3
C5B—C4B—C3B	121.0 (3)	C27—C26—H26A	121.3
C5B—C4B—H4B	119.5	C28—C27—C26	122.7 (4)
C3B—C4B—H4B	119.5	C28—C27—H27A	118.6
C4B—C5B—C6B	118.9 (2)	C26—C27—H27A	118.6
C4B—C5B—H5B	120.6	C27—C28—C29	118.8 (4)
C6B—C5B—H5B	120.6	C27—C28—H28A	120.6
C5B—C6B—C1B	119.1 (2)	C29—C28—H28A	120.6
C5B—C6B—C7B	134.2 (2)	C30—C29—C28	118.9 (4)
C1B—C6B—C7B	106.6 (2)	C30—C29—H29A	120.6
C8B—C7B—C12B	119.1 (2)	C28—C29—H29A	120.6
C8B—C7B—C6B	134.4 (2)	C25—C30—C29	121.3 (4)
C12B—C7B—C6B	106.5 (2)	C25—C30—H30A	119.4
C7B—C8B—C9B	120.4 (2)	C29—C30—H30A	119.4
			
C12A—N1A—C1A—C2A	176.3 (2)	C4B—C5B—C6B—C1B	0.5 (3)
C22A—N1A—C1A—C2A	−4.7 (4)	C4B—C5B—C6B—C7B	−176.9 (2)
C12A—N1A—C1A—C6A	−1.4 (2)	C2B—C1B—C6B—C5B	−0.4 (3)
C22A—N1A—C1A—C6A	177.6 (2)	N1B—C1B—C6B—C5B	−179.32 (19)
N1A—C1A—C2A—C3A	−177.1 (2)	C2B—C1B—C6B—C7B	177.7 (2)
C6A—C1A—C2A—C3A	0.4 (4)	N1B—C1B—C6B—C7B	−1.3 (2)
C1A—C2A—C3A—C4A	−1.8 (4)	C5B—C6B—C7B—C8B	−0.7 (4)
C2A—C3A—C4A—C5A	1.4 (4)	C1B—C6B—C7B—C8B	−178.4 (2)
C3A—C4A—C5A—C6A	0.6 (4)	C5B—C6B—C7B—C12B	178.0 (2)
C4A—C5A—C6A—C1A	−2.0 (4)	C1B—C6B—C7B—C12B	0.4 (2)
C4A—C5A—C6A—C7A	176.8 (2)	C12B—C7B—C8B—C9B	−0.9 (3)
N1A—C1A—C6A—C5A	179.5 (2)	C6B—C7B—C8B—C9B	177.7 (2)
C2A—C1A—C6A—C5A	1.6 (3)	C7B—C8B—C9B—C10B	1.6 (3)
N1A—C1A—C6A—C7A	0.4 (2)	C7B—C8B—C9B—C13B	−178.2 (2)
C2A—C1A—C6A—C7A	−177.5 (2)	C8B—C9B—C10B—C11B	−1.2 (4)
C5A—C6A—C7A—C8A	2.7 (4)	C13B—C9B—C10B—C11B	178.6 (2)
C1A—C6A—C7A—C8A	−178.4 (2)	C9B—C10B—C11B—C12B	0.0 (4)
C5A—C6A—C7A—C12A	−178.2 (3)	C1B—N1B—C12B—C11B	178.5 (2)
C1A—C6A—C7A—C12A	0.7 (2)	C22B—N1B—C12B—C11B	8.5 (4)
C12A—C7A—C8A—C9A	1.7 (3)	C1B—N1B—C12B—C7B	−1.4 (2)
C6A—C7A—C8A—C9A	−179.3 (2)	C22B—N1B—C12B—C7B	−171.4 (2)
C7A—C8A—C9A—C10A	0.3 (3)	C10B—C11B—C12B—N1B	−179.2 (2)
C7A—C8A—C9A—C13A	−178.8 (2)	C10B—C11B—C12B—C7B	0.7 (3)
C8A—C9A—C10A—C11A	−1.6 (4)	C8B—C7B—C12B—N1B	179.6 (2)
C13A—C9A—C10A—C11A	177.5 (2)	C6B—C7B—C12B—N1B	0.6 (2)
C9A—C10A—C11A—C12A	0.8 (4)	C8B—C7B—C12B—C11B	−0.3 (3)
C1A—N1A—C12A—C11A	−177.9 (2)	C6B—C7B—C12B—C11B	−179.3 (2)
C22A—N1A—C12A—C11A	3.1 (4)	C14B—N2B—C13B—C9B	−180.0 (2)
C1A—N1A—C12A—C7A	1.9 (2)	C8B—C9B—C13B—N2B	176.5 (2)
C22A—N1A—C12A—C7A	−177.1 (2)	C10B—C9B—C13B—N2B	−3.3 (4)
C10A—C11A—C12A—N1A	−179.0 (2)	C13B—N2B—C14B—C15B	−177.0 (2)
C10A—C11A—C12A—C7A	1.3 (3)	C13B—N2B—C14B—S1B	2.7 (3)
C8A—C7A—C12A—N1A	177.68 (19)	C21B—S1B—C14B—N2B	179.4 (2)
C6A—C7A—C12A—N1A	−1.6 (2)	C21B—S1B—C14B—C15B	−0.92 (19)
C8A—C7A—C12A—C11A	−2.5 (3)	N2B—C14B—C15B—C16B	−179.5 (2)
C6A—C7A—C12A—C11A	178.2 (2)	S1B—C14B—C15B—C16B	0.9 (3)
C14A—N2A—C13A—C9A	175.7 (2)	N2B—C14B—C15B—C24B	0.9 (4)
C8A—C9A—C13A—N2A	8.2 (4)	S1B—C14B—C15B—C24B	−178.80 (19)
C10A—C9A—C13A—N2A	−170.9 (2)	C14B—C15B—C16B—C21B	−0.3 (3)
C13A—N2A—C14A—C15A	177.1 (2)	C24B—C15B—C16B—C21B	179.4 (2)
C13A—N2A—C14A—S1A	−4.6 (3)	C14B—C15B—C16B—C17B	178.6 (2)
C21A—S1A—C14A—C15A	0.84 (18)	C24B—C15B—C16B—C17B	−1.8 (4)
C21A—S1A—C14A—N2A	−177.6 (2)	C21B—C16B—C17B—C18B	−14.7 (5)
N2A—C14A—C15A—C24A	−1.4 (4)	C15B—C16B—C17B—C18B	166.5 (4)
S1A—C14A—C15A—C24A	−179.89 (19)	C21B—C16B—C17B—C18X	13.2 (5)
N2A—C14A—C15A—C16A	177.8 (2)	C15B—C16B—C17B—C18X	−165.6 (4)
S1A—C14A—C15A—C16A	−0.7 (2)	C16B—C17B—C18B—C19B	43.5 (8)
C14A—C15A—C16A—C21A	0.1 (3)	C18X—C17B—C18B—C19B	−31.3 (8)
C24A—C15A—C16A—C21A	179.3 (2)	C17B—C18B—C19B—C20B	−66.4 (10)
C14A—C15A—C16A—C17A	−177.5 (2)	C18B—C17B—C18X—C19X	66.9 (11)
C24A—C15A—C16A—C17A	1.7 (4)	C16B—C17B—C18X—C19X	−48.0 (10)
C21A—C16A—C17A—C18A	−16.2 (4)	C17B—C18X—C19X—C20B	61.3 (13)
C15A—C16A—C17A—C18A	161.2 (2)	C18X—C19X—C20B—C21B	−40.4 (10)
C16A—C17A—C18A—C19A	44.0 (4)	C18X—C19X—C20B—C19B	32.8 (6)
C17A—C18A—C19A—C20A	−58.4 (4)	C18B—C19B—C20B—C21B	50.9 (8)
C18A—C19A—C20A—C21A	39.9 (3)	C18B—C19B—C20B—C19X	−67.6 (9)
C15A—C16A—C21A—C20A	−176.8 (2)	C15B—C16B—C21B—C20B	178.8 (2)
C17A—C16A—C21A—C20A	0.9 (4)	C17B—C16B—C21B—C20B	−0.1 (4)
C15A—C16A—C21A—S1A	0.6 (3)	C15B—C16B—C21B—S1B	−0.4 (3)
C17A—C16A—C21A—S1A	178.22 (18)	C17B—C16B—C21B—S1B	−179.31 (19)
C19A—C20A—C21A—C16A	−11.9 (4)	C19X—C20B—C21B—C16B	16.1 (7)
C19A—C20A—C21A—S1A	171.02 (19)	C19B—C20B—C21B—C16B	−16.2 (4)
C14A—S1A—C21A—C16A	−0.81 (19)	C19X—C20B—C21B—S1B	−164.8 (5)
C14A—S1A—C21A—C20A	176.6 (2)	C19B—C20B—C21B—S1B	162.9 (3)
C12A—N1A—C22A—C23A	−90.5 (3)	C14B—S1B—C21B—C16B	0.8 (2)
C1A—N1A—C22A—C23A	90.7 (3)	C14B—S1B—C21B—C20B	−178.4 (2)
C12B—N1B—C1B—C2B	−177.2 (2)	C12B—N1B—C22B—C23B	89.1 (3)
C22B—N1B—C1B—C2B	−6.9 (4)	C1B—N1B—C22B—C23B	−79.4 (3)
C12B—N1B—C1B—C6B	1.7 (2)	C30—C25—C26—C27	−2.0 (7)
C22B—N1B—C1B—C6B	171.9 (2)	C25—C26—C27—C28	−1.3 (7)
N1B—C1B—C2B—C3B	178.7 (2)	C26—C27—C28—C29	2.8 (7)
C6B—C1B—C2B—C3B	0.0 (3)	C27—C28—C29—C30	−1.0 (6)
C1B—C2B—C3B—C4B	0.2 (4)	C26—C25—C30—C29	3.8 (6)
C2B—C3B—C4B—C5B	−0.1 (4)	C28—C29—C30—C25	−2.2 (6)
C3B—C4B—C5B—C6B	−0.3 (4)		

### Hydrogen-bond geometry (Å, °)

**Table:**

Cg1, Cg2 and Cg3 are the centroids of the C1B–C6B, C7A–C12A and C14A–C16A/C21A/S1A rings, respectively.

**Table:**

*D*—H···*A*	*D*—H	H···*A*	*D*···*A*	*D*—H···*A*
C22A—H22A···N3B^i^	0.97	2.59	3.487 (3)	155
C11A—H11A···Cg1^ii^	0.93	2.65	3.499 (3)	153
C11B—H11B···Cg2^i^	0.93	2.82	3.725 (3)	166
C27—H27A···Cg3^iii^	0.93	2.71	3.625 (6)	169

Symmetry codes: (i) −*x*+1, −*y*+2, −*z*; (ii) *x*−1, *y*, *z*; (iii) −*x*, −*y*+1, −*z*+1.
